# Prevalence and sociodemographic trends of weight misperception in Korean adolescents

**DOI:** 10.1186/1471-2458-14-452

**Published:** 2014-05-13

**Authors:** Seonho Kim, Wi-Young So

**Affiliations:** 1 Department of Nursing, Chungbuk National University, Cheongju, South Korea; 2 Department of Human Movement Science, Seoul Women’s University, Seoul, South Korea

**Keywords:** Adolescents, Overestimation, Prevalence, Underestimation, Weight misperception

## Abstract

**Background:**

Rapid physical and emotional growth occurs during youth. Adolescence is one of the most important periods for adapting to body change and establishing an ideal body image. Body change is an important and sensitive concern for adolescents, and the values and self-conception established at this time affect various aspects of the entire life. This study aimed to investigate the prevalence and trends of weight misperception among adolescents in Korea.

**Methods:**

We analyzed raw data from the 2011 Korea Youth Risk Behaviour Web-based Survey-VII (KYRBWS-VII), in which 73,474 adolescents from the middle-school first grade to the high-school third grade (aged 12–18) participated. For the multivariate logistic regression model, the dependent variable was existence of misperception (yes/no) and independent variables were sociodemographic factors.

**Results:**

We found that the prevalence of weight misperception was 49.3% (overestimation, 23.7%; underestimation, 25.6%). Among male students, 65.0% underestimated their weight, whereas 62.2% of female students overestimated their weight. Multivariate logistic regression analysis revealed that gender (OR = 1.254; 95% CI = 1.214–1.294; p < 0.001) and socioeconomic status (OR = 1.173; 95% CI = 1.121–1.228; p < 0.001) were significantly associated with weight misperception. Compared with overweight/obese adolescents, adolescents whose BMI-based body weight was underweight or normal showed 2.386-times higher (OR = 2.386; 95% CI = 2.045–2.783, p < 0.001) and 32.804-times higher (OR = 32.804; 95% CI = 29.829–36.077, p < 0.001) body shape misperception, respectively.

**Conclusions:**

An effective obesity intervention program for adolescents must reflect and monitor body shape perception as well as BMI, and should include subjects who overestimate as well as underestimate their weight.

## Background

Rapid physical and emotional growth occurs during youth. Adolescence is one of the most important periods for adapting to body change and establishing an ideal body image. Body change is an important and sensitive concern for adolescents, and the values and self-conception established at this time affect various aspects of the entire life [1,2].

People tend to regard low weight as normal and prefer a thin body type [1]. Dissatisfaction with one’s body shape and extreme dieting have become common because the trend in public opinion and mass media is to lay emphasis on slimness and appearance [3-5]. Under this influence, an adolescent whose ego is not established recognizes a thin body shape as the standard of beauty, and this attitude distorts the perception of and increases dissatisfaction with their body shape [6], causing bad eating habits and excessive reduction in body weight [3,7-9].

Misperception of the ideal body shape threatens the physical and mental health of adolescents during growth. Obese, normal, and even underweight individuals may try immoderate weight control measures and experience depression [8,10-14] that extends even into the early adult period [15]. Misperception of body shape is therefore a serious public health problem and negatively affects the future as well as present physical and mental health in adolescents.

Known differences exist between male and female students in perception of body shape. While female students tend to overestimate their weight, male students tend to underestimate their weight [1,14,16-23]. Female students also have more interest in body weight [7,18] and control their body weight more aggressively than do male students [3,17,19,24]. Furthermore, male students tend to underestimate their body weight even though their weight is normal, because they have more interest in increasing muscle mass [25].

In Western countries, many studies have been conducted on current attitudes and sociodemographic factors that contribute to misperception of body shape in order to provide a basis for effective adolescent weight control programs [9,18,26-29]. However, Korean studies on misperception of body shape in adolescents examined mainly the relationship between perception of body shape and eating habits, self-esteem, and depression [1,14,20,30-35], ignoring factors affecting discordance. The adolescents who regarded their body shape as normal or thin even though they were overweight or obese had a lower participation rate in weight control programs, because they lacked the need of weight control [18,36]. Nevertheless, subjects in most domestic studies were overweight/obese adolescents; adolescents who underestimated their weight were rarely included. It was also difficult to generalize the findings because most domestic studies included adolescents living in a single specific region [1,20,30,34].

The present study aimed to identify the factors affecting misperception of body shape in adolescents underestimating and overestimating their body weight and to provide evidence for future obesity-management policies and interventions. Specifically, we addressed (1) the present condition of misperception by comparing perception of body shape and weight status, and (2) sociodemographic characteristics affecting discordance between perception of body shape and weight status (overestimation and underestimation).

## Methods

### Design

This study is a secondary analysis of raw data from The 7th Korea Youth Risk Behavior web-based Survey (KYRBWS-VII), conducted in 2011 by the Korea Centers for Disease Control and Prevention in middle and high schools throughout the country.

### Original data & samples

The KYRBWS-VII is self-reported anonymous online survey conducted by the Ministry Of Education, Science And Technology, Ministry Of Health And Welfare, and Korea Centers For Disease Control And Prevention. This survey was conducted for students from first grade in middle school (7th) to third grade in high school (12th) (aged 12–18 years) in order to assess the health behavior of Korean adolescents. The subjects of KYRBWS-VII were 79,202 middle and high school students registered in 800 schools in April 2010; subjects were selected through stratification, allocation, and extraction sampling. Of these, 75,643 (95.5%) completed this study. An additional 2,169 students who did not provide weight and height information were excluded, leaving a total of 73,474 students in the final analysis.

### Measurements

The KYRBWS-VII consisted of 99 indicators in 14 domains, including smoking, drinking, obesity and weight control, eating habits, and physical activity. The items and indicators were proposed by an advisory committee in each area, based on domestic and foreign data.

The following variables were evaluated. General characteristics of subjects included gender, class, region, residential type, and socioeconomic status (SES). Class was divided into middle and high school categories, and region was divided into district, rural city, and metro city categories. Residential type was divided into living with family and other categories, and socioeconomic status was divided into low, middle, and high categories.

Subjective body shape perception (BSP) was assessed using the response to the following question: “How do you think your body shape is?” In the underweight group, the responses were “very thin” and “slightly thin”; in the normal weight group, “normal”; and in the overweight/obesity group, “slightly fat” and “very fat.” Self-reported height and weight were used to calculate body mass index (BMI). BMI was classified using the 2007 Korea growth chart for children and adolescents. Subjects below the 5th percentile in each age group were included in the underweight group. Subjects between the 5th and 84th percentile in each age group were included in the normal weight group, and subjects above the 85th percentile in each age group were included in the overweight/obesity group [37]. Misperception of body shape was classified as concordant when weight status according to BMI agreed with subjective BSP and discordant when it disagreed. For the multivariate logistic regression model, the dependent variable was existence of misperception (yes/no) and independent variables were sociodemographic factors.

### Data collection

KYRBWS-VII was conducted for 45–50 minutes during class in the computer room at Internet-enabled schools. A teacher assigned students to a computer and documented student visits to the home page using an assigned id number. Ethical approval was not required because the survey did not collect private information such as name, school, address, phone number, social security number, and presence of any disease. Furthermore, all study procedures were approved by the Ministry Of Education, Science And Technology; Ministry Of Health And Welfare; and Korea Centers For Disease Control And Prevention on March 2012.

### Statistical methods

Differences in the misperception rate of body shape were calculated on the basis of general characteristics, and the chi-square test was used to analyze resulting frequency data. Multivariate logistic regression analysis was used to identify factors affecting misperception of body shape. Data analysis was performed using SPSS Complex Sample™ version 18.0 (SPSS, Chicago, IL, USA).

## Results

### General characteristics of the subjects

The distributions of female students, middle school students, and those living in metro cities were 50.0%, 50.9%, and 45.9% respectively. The distributions of adolescents living with family and those with a SES of “middle”, which is the most prevalent, were 95.3% and 47.6%, respectively. The distributions of adolescents who regarded their weight as normal, overweight/obese, and underweight were 34.2%, 37.2%, and 28.6%, respectively. The distributions of adolescents who were normal weight, overweight/obese, and underweight according to BMI were 81.6%, 14.4%, and 4.0%, respectively (Table 1).

**Table 1:** Characteristics of the study sample and distribution of weight perception within each BMI-based weight status

**Classification**	**Total**		**Male**		**Female**	
	**n**	**(%)**	**n**	**(%)**	**n**	**(%)**
Gender						
Male	36,755	(50.0)	-	-	-	-
Female	36,719	(50.0)	-	-	-	-
School						
Middle	37,425	(50.9)	19,007	(51.7)	18,418	(50.2)
High	36,049	(49.1)	17,748	(48.3)	18,301	(49.8)
Region						
Rural	9,501	(12.9)	4,930	(13.4)	4,571	(12.4)
City	30,236	(41.2)	14,825	(40.3)	15,411	(42.0)
Metro city	33,737	(45.9)	17,000	(46.3)	16,737	(45.6)
Living with family						
Yes	70,005	(95.3)	34,947	(95.1)	35,058	(95.5)
No	3,469	(4.7)	1,808	(4.9)	1,661	(4.5)
SES (socioeconomic status)						
High	21,767	(29.6)	12,184	(33.1)	9,583	(26.1)
Middle	34,982	(47.6)	16,535	(45.0)	18,447	(50.2)
Low	16,725	(22.8)	8,036	(21.9)	8,689	(23.7)
Weight perception						
Underweight	21,039	(28.6)	13,283	(36.1)	7,756	(21.1)
Right weight	25,116	(34.2)	11,601	(31.6)	13,515	(36.8)
Overweight/obese	27,319	(37.2)	11,871	(32.3)	15,448	(42.1)
BMI based weight status						
Underweight	2,947	(4.0)	1,431	(3.9)	1,516	(4.1)
Normal weight	59,943	(81.6)	29,650	(80.7)	30,293	(82.5)
Overweight/obese	10,584	(14.4)	5,674	(15.4)	4,910	(13.4)
Total	73,474	(100.0)	36,755	(100.0)	36,719	(100.0)

### Misperception rate of body shape

The comparison between self-recognized and BMI-based weight status is summarized in Table 2. Table 3 shows the comparison of self-recognized weight status with BMI-based weight status according to sociodemographic characteristics. Misperception rate was significantly higher in male subjects (51.1%) than in female subjects (47.5%) (p < 0.001) and in high school students (50.0%) than in middle school students (48.6%) (p < 0.001). Female high school students had a significantly higher misperception rate (48.5%) than middle school students (46.5%) (p < 0.001). However, male students did not show any significant difference (p = 0.070). Perception of weight was significantly related to SES and BMI-based weight status as adolescents with low SES (51.5%) (p < 0.001) and BMI-based normal weight (59.2%) (p < 0.001) had a higher misconception than did adolescents in other groups.

**Table 2:** Distribution of weight perception according to each BMI based weight status (unit: n, %)

**BMI based weight status**	**Weight perception**						**Total**	
	**Underweight**		**Normal weight**		**Overweight/obese**			
Total								
Underweight	2,662	(90.3)	230	(7.8)	55	(1.9)	2,947	(100.0)
Normal weight	18,335	(30.6)	24,469	(40.8)	17,139	(28.6)	59,943	(100.0)
Overweight/obese	42	(0.4)	417	(3.9)	10,125	(95.7)	10,584	(100.0)
Male								
Underweight	1,323	(92.5)	90	(6.3)	18	(1.3)	1,431	(100.0)
Normal weight	11,925	(40.2)	11,258	(38.0)	6,467	(21.8)	29,650	(100.0)
Overweight/obese	35	(0.6)	253	(4.5)	5,386	(94.9)	5,674	(100.0)
Female								
Underweight	1,339	(88.3)	140	(9.2)	37	(2.4)	1,516	(100.0)
Normal weight	6,410	(21.2)	13,211	(43.6)	10,672	(35.2)	30,293	(100.0)
Overweight/obese	7	(0.1)	164	(3.3)	4,739	(96.5)	4,910	(100.0)

**Table 3:** Percentage distribution of concordant versus discordant weight perception (unit: %)

**Classification**	**Total**			**Male**			**Female**		
	**Weight perception**		**χ**^**2 **^**( *p *)**	**Weight perception**		**χ**^**2 **^**( *p *)**	**Weight perception**		**χ**^**2 **^**( *p *)**
	**Concordant**	**Discordant**		**Concordant**	**Discordant**		**Concordant**	**Discordant**	
Gender									
Male	48.9	51.1	97.811	-	-		-	-	
Female	52.5	47.5	(<0.001)	-	-		-	-	
School									
Middle	51.4	48.6	14.519	49.3	50.7	3.284	53.5	46.5	14.434
High	50.0	50.0	(<0.001)	48.4	51.6	(0.070)	51.5	48.5	(<0.001)
Region									
Rural	51.0	49.0	2.021	48.4	51.6	8.091	53.8	46.2	4.232
City	50.4	49.6	(0.364)	48.1	51.9	(0.018)	52.6	47.4	(0.121)
Metro city	50.9	49.1		49.7	50.3		52.1	47.9	
Living with family									
Yes	50.7	49.3	0.310	48.9	51.1	0.783	52.5	47.5	0.050
No	50.2	49.8	(0.578)	47.9	52.1	(0.390)	52.8	47.2	(0.823)
SES (Socioeconomic status)									
High	50.4	49.6	52.495	49.4	50.6	14.010	51.8	48.2	45.367
Middle	51.9	48.1	(<0.001)	49.4	50.6	(<0.001)	54.1	45.9	(<0.001)
Low	48.5	51.5		47.0	53.0		49.9	50.1	
BMI-based weight status									
Underweight	90.3	9.7	12753.171	92.5	7.5	7313.878	88.3	11.7	5555.263
Normal	40.8	59.2	(<0.001)	38.0	62.0	(<0.001)	43.6	56.4	(<0.001)
Overweight/obese	95.7	4.3		94.9	5.1		96.5	3.5	
Total	50.7	49.3		48.9	51.1		52.5	47.5	

Concordant, discordant (overweight, underweight).

Tested by chi-square analysis.

Table 4 shows the results of misperception of body shape by overestimation vs. underestimation. Male students showed significantly higher underestimation (65.0%), and female students showed significantly higher overestimation (62.2%) (p < 0.001). Middle school students showed significantly higher underestimation (55.7%), and high school students showed significantly higher overestimation (51.9%) (p < 0.001). The type of discordance was significantly related to SES and BMI-based weight status as adolescents in the low SES group (52.5%) (p < 0.001) and BMI-based underweight group (100.0%) (p < 0.001) had higher overestimation than that of the other groups.

**Table 4:** Percentage distribution of overestimation and underestimation in discordant weight perception (unit: %)

**Classification**	**Total**			**Male**			**Female**		
	**Weight perception**		**χ**^**2 **^**( *p *)**	**Weight perception**		**χ**^**2 **^**( *p *)**	**Weight perception**		**χ**^**2 **^**( *p *)**
	**Overestimation**	**Underestimation**		**Overestimation**	**Underestimation**		**Overestimation**	**Underestimation**	
Gender									
Male	35.0	65.0	2688.990	-	-		-	-	
Female	62.2	37.8	(<0.001)	-	-		-	-	
School									
Middle	44.3	55.7	210.612	35.2	64.8	0.218	54.6	45.4	416.070
High	51.9	48.1	(<0.001)	34.8	65.2	(0.641)	69.6	30.4	(<0.001)
Region									
Rural	46.7	53.3	4.497	34.4	65.6	2.980	61.5	38.5	1.178
City	48.2	51.8	(0.100)	34.5	65.5	(0.225)	62.7	37.3	(0.555)
Metro city	48.4	51.6		35.7	64.3		62.0	38.0	
Living with family									
Yes	48.1	51.9	0.046	35.1	64.9	2.305	62.1	37.9	5.119
No	47.9	52.1	(0.830)	32.7	67.3	(0.129)	66.1	33.9	(0.024)
SES									
High	43.6	56.4	157.650	35.0	65.0	2.194	55.0	45.0	181.041
Middle	48.8	51.2	(<0.001)	34.5	65.5	(0.334)	62.8	37.2	(<0.001)
Low	52.5	47.5		35.9	64.1		68.7	31.3	
BMI-based weight status									
Underweight	100.0	0.0	733.550	100.0	0.0	355.880	100.0	0.0	389.658
Normal	48.3	51.7	(<0.001)	35.2	64.8	(<0.001)	62.5	37.5	(<0.001)
Overweight/obese	0.0	100.0		0.0	100.0		0.0	100.0	
Total	48.1	51.9		35.0	65.0		62.2	37.8	

Tested by chi-square analysis.

### Factors affecting misperception of body shape

Gender, SES, and BMI-based weight status affected misperception of body shape across all groups. Misperception of body shape was 1.254-times higher in male subjects (95% CI = 1.214–1.294, p < 0.001) and 1.173-times higher in adolescents with high socioeconomic status (95% CI = 1.121–1.228, p < 0.001). Compared with overweight/obese adolescents, adolescents whose BMI-based body weight was underweight or normal showed 2.386-times higher (95% CI = 2.045–2.783, p < 0.001) and 32.804-times higher (95% CI = 29.829–36.077, p < 0.001) body shape misperception, respectively.

Female high school students showed 1.062-times higher misperception than their middle school counterparts (95% CI = 1.014–1.111; p = 0.010). The remaining results were similar with those from the total adolescent population (Table 5).

**Table 5:** Multivariate logistic analysis of factors affecting misperception of weight

**Classification**	**Total**			**Male**			**Female**		
	**OR**	**95% CI**	***p***	**OR**	**95% CI**	***p***	**OR**	**95% CI**	***p***
Gender									
Female	1.000								
Male	1.254	1.214-1.294	<0.001						
School									
Middle	1.000			1.000			1.000		
High	1.026	0.993-1.060	0.122	0.958	0.940-1.032	0.529	1.062	1.014-1.111	0.010
Region									
City	1.000			1.000			1.000		
Rural	0.979	0.930-1.030	0.416	0.974	0.906-1.047	0.470	0.985	0.916-1.060	0.691
Metro city	0.980	0.947-1.014	0.249	0.958	0.912-1.006	0.087	1.001	0.954-1.050	0.981
Living with family									
Yes	1.000			1.000			1.000		
No	1.070	0.992-1.154	0.080	1.074	0.966-1.193	0.186	1.063	0.953-1.186	0.271
SES									
High	1.000			1.000			1.000		
Middle	0.983	0.903-1.073	0.724	0.973	0.923-1.025	0.304	0.090	0.862-0.959	<0.001
Low	1.173	1.121-1.228	<0.001	1.125	1.055-1.200	<0.001	1.221	1.144-1.302	<0.001
BMI-based weight status									
Overweight/obese	1.000			1.000			1.000		
Underweight	2.386	2.045-2.783	<0.001	1.519	1.208-1.911	<0.001	3.692	2.965-4.596	<0.001
Normal	32.804	29.829-36.077	<0.001	30.634	27.146-34.571	<0.001	36.640	31.396-42.760	<0.001

OR = Odds ratio; CI = Confidence interval.

Tested by multivariate logistic regression analysis.

## Discussion

The perception of body weight by adolescents affects eating habits, eating behavior, and weight control [9]. Misperception of body shape is important because it negatively affects future as well as present physical and mental health, such as occurrence of depression in young adulthood [8,10-15]. This study examined sociodemographic factors affecting misperception of body shape in Korean adolescents using data from the entire country, and attempted to provide basic information for future policies and education on adolescent obesity.

Only 50.7% of adolescents in our study correctly recognized their body weight. This value was lower than the 56.7–77.7% reported in other domestic studies [1,14,20] and the 70.1–77.7% suggested by studies from Western countries such as the United States and Australia [17,18,36,38]. Differences in subject characteristics, including height and weight, may account for these disparate results. Generally, in surveys including height and weight, BMI tends to be lower than the actual value because people report greater height and lower weight [18,39]. Thus, in a study using the survey, the accuracy of weight perception is lowered by large difference between actual BMI and weight perception; therefore, the study using the survey would have lower accuracy of weight perception than that of other studies using actual measurements. Compared with American, Chinese, and Japanese adolescents, Korean adolescents are more sensitive to appearance, have more interest in weight control, and exhibit more weight-related behavior [40].

This result indicated that because of different cultural pressures, Korean adolescents are facing social environments that could predispose them to higher rates of weight misconception. Furthermore, well-designed studies are necessary to solve this issue in Korean adolescents. It is also interesting that compared with Western students, Eastern students had a higher misperception of body shape. Zhang et al. [26] reported that compared to American adolescents, adolescents in Hong Kong, Macau, and Taipei had a higher rate of body shape misperception, and that the perception of body shape is affected by sociocultural factors such as region and race [17,18,26,41-43]. Additional studies are required to include Korean adolescents as well as Western students from the Americas and Europe.

Our findings that male students tend to underestimate their body weight and female students tend to overestimate their body weight are consistent with findings of previous studies [1,14,16-23]. While 29.5% of female students overestimated their weight, only 19.7% underestimated their weight. Overestimation promotes weight control by causing dissatisfaction with body shape [3,6-9,44]. However, it tends to quickly reduce body weight by unhealthy weight control behavior such as drug usage, fasting, smoking, and single-food diets [3,7,18,35,45]. Previous studies showed that 13.5–22.9% of Korean adolescents trying to control body weight showed unhealthy weight control behavior, a higher level than that found in China, the Americas, and Japan [39,40]. This suggests that intensive management is required for adolescents who overestimate their weight.

In contrast, male students showed higher rate of underestimation (33.2%) compared with overestimation (17.9%). This rate is higher than the 9.8% reported by Park in American adolescents [18]. Previous studies on perception of body shape focused on adolescents overestimating their weight [1,8,21,28,36]. The present study suggests that adolescents underestimating their body weight also require attention and study. Weight control is motivated by self-perception of body shape and not true weight [3]; these adolescents have lower motivation, resulting in many problems such as ineffectiveness of preventive interventions for obesity and eating disorder behaviors [6,18].

Actual weight was the most important factor affecting misperception. Interestingly, adolescents who had a normal weight had a higher misperception rate than did overweight/obese adolescents. The misperception rate was 32.8-times higher in normal weight students compared with obese students, implying that normal weight students as well as obese students should be considered in weight control programs.

Future obesity intervention programs should include normal-weight adolescents having different subjective perceptions of body shape. In addition, obesity policies for adolescents should include monitoring the subjective perception of body shape as well as actual BMI.

The lower the SES, the higher is the misperception of body shape in adolescents. This suggests that adolescents at a lower SES level should be the subjects of obesity programs, because the obesity rate is high in this group [25]. Class does not affect discordance in total student analysis, but female high school students showed higher misperception than their middle school counterparts. This result corresponds to results of previous studies [18,30]. High school female students show a higher diet stress than do middle school females [46]. This is thought to represent higher interest in body shape in high school students, because they have a shorter time for adjusting to social interactions such as finding employment or entering a university/college.

This study has several limitations. First, we could determine interrelationships alone and not cause and effect because this was a retrospective and cross-sectional study. Second, the height and weight were self-reported, which limits accuracy. In general, compared with male students, female students tend to report lower height and weight than the actual value [47]. This tendency may account for the different results between male and female students. However, studies based on large samples actually use self-reported height and weight; furthermore, actual height and weight were assumed to be recorded as this study also was a self-reported survey performed using a computer. In addition, a previous study suggested that little discrepancy was found between self-reported and measured BMI in Asian adolescents [48]. Nevertheless, in future large studies, height and weight should be directly measured to improve assessment of weight perception. Third, factors affecting misperception of weight were limited to sociodemographic variables, limiting the evaluation of overall factors. Future studies should include physical and mental variables. In spite of these limitations, the findings of this study are significant because notional data representing the entire Korean adolescent population were investigated for the first time, and the discordance between perception of body shape and actual weight was determined by examining both overestimation and underestimation. In addition, the results of this study can facilitate selection of subjects for adolescent obesity programs by proposing that both BMI-based weight status and subjective perception of body shape should be monitored and reflected.

## Conclusions

In conclusion, 50.7% of Korean adolescents correctly recognized their body shape, while 33.2% of male students underestimated and 29.6% of female students overestimated their weight. In the overall analysis of adolescents, gender, socioeconomic status, and BMI-based weight status affected misperception of body shape. Class was an additional factor in case of female students. An effective obesity program should monitor and reflect both BMI-based weight status and subjective perception of body shape in adolescents who underestimate or overestimate their weight.

## Competing interests

The author has no competing interests.

## Authors’ contributions

Both S K and W-Y S contributed to the study design and management, performed statistical analyses, and drafted the manuscript. Both authors read and approved the final manuscript.

## Pre-publication history

The pre-publication history for this paper can be accessed here:

http://www.biomedcentral.com/1471-2458/14/452/prepub
